# Bone-Forming Peptide-4 Induces Osteogenic Differentiation and VEGF Expression on Multipotent Bone Marrow Stromal Cells

**DOI:** 10.3389/fbioe.2021.734483

**Published:** 2021-10-06

**Authors:** Mi Eun Kim, Jong Keun Seon, Ju Yeon Kang, Taek Rim Yoon, Jun Sik Lee, Hyung Keun Kim

**Affiliations:** ^1^ Department of Biology, Immunology Research Lab, Integrative Biological Sciences & BK21 FOUR Educational Research Group for Age-Associated Disorder Control Technology, College of Natural Sciences, Chosun University, Gwangju, South Korea; ^2^ Korea Biomedical Materials and Devices Innovation Research Center of Chonnam National University Hospital, Gwangju, South Korea; ^3^ Department of Orthopaedics Surgery, Center for Joint Disease of Chonnam National, University Hwasun Hospital, Jeonnam, South Korea

**Keywords:** bone morphogenetic proteins, bone-forming peptides, osteogenic differentiation, bone formation, VEGF

## Abstract

Bone morphogenetic proteins (BMPs) have been widely used as treatment for bone repair. However, clinical trials on fracture repair have challenged the effectiveness of BMPs and suggested that delivery of multipotent bone marrow stromal cells (BMSCs) might be beneficial. During bone remodeling and bone fracture repair, multipotent BMSCs differentiate into osteoblasts or chondrocytes to stimulate bone formation and regeneration. Stem cell-based therapies provide a promising approach for bone formation. Extensive research has attempted to develop adjuvants as specific stimulators of bone formation for therapeutic use in patients with bone resorption. We previously reported for the first time bone-forming peptides (BFPs) that induce osteogenesis and bone formation. BFPs are also a promising osteogenic factor for prompting bone regeneration and formation. Thus, the aim of the present study was to investigate the underlying mechanism of a new BFP-4 (FFKATEVHFRSIRST) in osteogenic differentiation and bone formation. This study reports that BFP-4 induces stronger osteogenic differentiation of BMSCs than BMP-7. BFP-4 also induces ALP activity, calcium concentration, and osteogenic factors (Runx2 and osteocalcin) in a dose dependent manner in BMSCs. Therefore, these results indicate that BFP-4 can induce osteogenic differentiation and bone formation. Thus, treatment of multipotent BMSCs with BFP-4 enhanced osteoblastic differentiation and displayed greater bone-forming ability than BMP-7 treatment. These results suggest that BFP-4-stimulated cell therapy may be an efficient and cost-effective complement to BMP-7-based clinical therapy for bone regeneration and formation.

## Introduction

Bone is a constantly renewing tissue by a process that is exquisitely balanced by bone-forming cells, bone-resorbing cells, osteoblasts, and osteoclasts (Douglas et al., 2018; Hamamura et al., 2019; Vesela et al., 2019). Therefore, achieving the proper balance between osteoclastic bone resorption and osteoblastic bone formation is important to prevent osteoporosis (Lerner et al., 2019; Yin et al., 2019). Moreover, bone remodeling is strongly regulated by interactions among cytokines, hormones, and other molecules that affect communication between osteoclasts and osteoblasts. Any disruption in this network may result in abnormal bone mass, including osteoporosis (Florencio-Silva et al., 2015; Han et al., 2018; Owen and Reilly, 2018; Kumar and Roger, 2019).

Various materials for osteogenesis and bone formation have been studied for a long time, and bone morphogenetic proteins (BMPs) is a representative factor with good efficacy in bone formation. Osteogenesis is controlled by multiple factors, including BMPs, and stromal cell-derived factors (SDFs) (Yang F. et al., 2018; Kim et al., 2018). BMPs are the key proteins mediating the differentiation, recruitment, and maturation of mesenchymal stromal cells (MSCs) into osteoblasts via osteogenesis (Sangadala et al., 2019). BMP-7 has been permitted for clinical use in the regeneration of bone in vertebral arthrodesis and fracture healing (Shen et al., 2010). According to previous reports, BMP-7 demonstrated a synergistic effect with microfractures to stimulate cartilage repair *in vivo*. However, some reports induced adipogenesis instead of bone/cartilage differentiation. Therefore, it is still required to find a more stable and economical inducer than BMP-7 as a factor inducing bone formation.

Several signaling pathways play important roles in regulating osteogenesis. BMPs, as a group, are considered one of the strongest osteoinductive factors. The biological activity of BMP-7 is active in the mature region of BMP-7. In contrast, bone-forming peptides (BFPs) is peptide found in the immature precursor of BMP-7. BFPs has a smaller molecular size and is more economical than BMP-7, suggesting the possibility of using it as a new therapeutic agent for bone formation. We previously reported that BFPs, including BFP-1, BFP-2, and BFP-3, are some of the most potent BMPs in inducing osteogenic differentiation (Kim et al., 2012; Kim et al., 2017; Lee et al., 2018). They have been shown to effectively induce osteogenic differentiation of multipotent bone marrow stromal cells (BMSCs) by regulating a panel of important downstream targets and through cross-talk with other important signaling pathways. In the case of BFP-1, we not only found the first reported peptide sequence with osteogenesis effect from the prodomain region of BMP-7, but also confirmed, through animal experiments, that BFP-1 induced bone formation by osteogenic differentiation more strongly than BMP-7. Furthermore, BFP-2 and BFP-3, both found in the prodomain region of BMP-7, have also been shown to have an osteogenic effect. It was reported that BFPs can replace BMP-7 and can be used for economically superior bone resorption-related disease therapies.

Therefore, we investigate to find a new BFP peptide that has more osteogenic effects than BMP-7. We isolated a new peptide sequences with osteogenic activity form the immature region of BMP-7 and investigated its osteogenic effects in multipotent BMSCs.

## Materials and Methods

### Chemical and Reagents

3-(4,5-dimethylthiazol-2-yl)-2,5-diphenyltetrazolium bromide (MTT), phosphate-buffered saline (PBS), dimethylsulfoxide, ethanol, Alizarin red Staining solution, Triton X-100, bovine serum albumin, and paraformaldehyde were purchased from Sigma-Aldrich (St. Louis, MO, United States).

### Synthesis and Purification of BFP-4

BFP-4 (FFKATEVHFRSIRST) was synthesized by Fmoc solid-phase peptide synthesis using an ASP48S automated peptide synthesizer (Peptron, Daejeon, South Korea) and purified by reverse-phase high-performance liquid chromatography using a Vydac Everest C18 column (250 mm × 22 mm, 10 μm) (UVISON, Sevenoaks, United Kingdom). Elution was conducted with a water-acetonitrile linear gradient (3–40% (v/v) acetonitrile) containing 0.1% (v/v) trifluoroacetic acid. The molecular mass of the purified peptide was confirmed by liquid chromatography/mass spectroscopy using an Agilent HP1100 series HPLC system (Santa Clara, CA, United States).

### Osteogenic Differentiation

Mouse multipotent bone marrow stromal cells were purchased from the American Type Culture Collection (ATCC, Manassas, VA, United States) and maintained in Dulbecco’s Modified Eagle Medium (DMEM) containing 10% fetal bovine serum (FBS) (Life Technologies, Grand Island, NY, United States). The multipotent BMSCs were seeded at a density of 1 × 10^4^ cells/well and maintained in culture for 3 days in a humidified atmosphere of 5% CO_2_ at 37°C. Experiments were performed after the cells had reached approximately 80% confluency. The culture medium was changed at day 3 to osteogenic differentiation medium (ODM; DMEM supplemented with 50 μg/ml ascorbic acid, 10 nM dexamethasone, and 10 mM β-glycerophosphate; all from Sigma-Aldrich, St. Louis, MO, United States) to induce osteogenic differentiation. After culture for 3 more days, one group of cells was cultured in ODM alone and a second group was cultured in ODM containing BFP-4 (0.01 and 0.1 μg/ml) and BMP-7 (0.01 and 0.1 μg/ml) (Kim et al., 2017; Geng et al., 2019).

### Cell Viability Assay

Surviving cells were counted using an MTT assay. Final concentration of 0.5 mg/ml MTT (17.2 mM phosphate-buffered saline (PBS), pH 6.5) was added to each well, and the plates were incubated for an additional 3 h. The solution was removed from the wells, and dimethylsulfoxide/ethanol (1:1 ratio) was added to dissolve the formazan products. The plates were shaken for 20 min, and the absorbance at 570 nm was recorded on a microplate spectrophotometer.

### Alizarin Red S Staining

Cell cultures were washed twice with distilled water, fixed for 1 h in ice-cold 70% ethanol, and rinsed twice with deionized water. Cultures were stained for 10 min with 1% Alizarin red S, and excess dye was removed by gently flushing with running water. Calcium deposits, which appeared bright red, were identified by light microscopy and photographed. Osteogenic differentiation was quantified by determining the density and area of Alizarin red S-stained regions with an image analysis program (Multi Gauge V3.0, Fujifilm, Tokyo, Japan).

### Calcium Assays

Calcium was assayed with the QuantiChrom Calcium Assay Kit (Gentaur, Voortstraat, Belgium). The calcium concentration was determined based on the formation of a stable blue complex between the phenolsulfonphthalein dye and free calcium, whereby color intensity was directly proportional to the concentration of free calcium in the sample. The cell layers were washed twice with PBS. Calcium in the matrix was dissolved with 0.5 M HCL, assay reagent was added. The color intensity was measured at 612 nm using an Infinite M200 microplate reader (Tecan, Männedorf, Switzerland).

### Reverse Transcription-Polymerase Chain Reaction

Total RNA was extracted from harvested BFP-4 treated BMSCs using PicoPure™ RNA Isolation Kit (ThermoFisher, USA) according to the manufacturer’s instruction. After isolation of RNA, reverse transcription was carried out using the SMARTer PCR cDNA synthesis Kit (Takara, Japan). One microgram RNA was used for the first-strand cDNA synthesis in a total volume of 20 μl according to the manufacturer’s instruction. RT-PCR was performed to assess the effects of BFP-4 on the transcription of the genes encoding alkaline phosphatase (ALP), osteocalcin, RUNX2, and the internal control housekeeping enzyme glyceraldehyde-3-phosphate dehydrogenase (GAPDH). The primers used were as follows: *ALP*, (forward) 5′-ACA CCT TGA CTG TGG TTA CTG CTG A-3′ and (reverse) 5′-CCT TGT AGC CAG GCC CGT TA-3′; *osteocalcin* (forward) 5ʹ-GAG GGC AAT AAG GTA GTG AAC AGA-3ʹ and (reverse) 5ʹ-AAG CCA TAC TGG TCT GAT AGC TCG-3ʹ; *Runx2*, (forward) 5ʹ-ACA AAC CAC AGA ACC ACA AGT-3ʹ and (reverse) 5ʹ-GTC TCG GTG GCT GGT AGT GA-3ʹ; and *GAPDH*, (forward) 5′-AAA TGG TGA AGG TCG GTG TG-3′ and (reverse) 5′-TGA AGG GGT CGT TGA TGG-3′ (Bioneer, Daejeon, South Korea). MSCs grown to 70% confluency on plates in the presence and absence of BFP-4 were homogenized in TRIzol reagent (Life Technologies). Total RNA was isolated and used to synthesize cDNA. To determine relative mRNA expression, house-keeping gene (GAPDH) and osteogenic differentiation marker gene with SYBR green I (SYBR advantage qPCR premix, Takara, Japan) were used.

### Immunofluorescence Analysis

In brief, a total of 1 × 10^4^ cells/well were seeded in an 6 well cell culture chamber slide containing completed medium for 3 days. The control group was assessed in the absence of osteogenic differentiation medium. At the end of 3 days incubation, the cells were treated by BFP-4 for 24 h. After 24 h incubation, cells were fixed with 4% paraformaldehyde prepared in PBS for 15 min, permeabilized with 0.1% Triton X-100 for 15 min, and blocked with 5% bovine serum albumin in PBS for 30 min. Coverslips were then incubated with a primary antibody against mouse CD44 and CD51 (eBioscience, San Diego, CA, United States) at a dilution of 1:200 followed by incubation with a secondary antibody at a dilution of 1:400, both at room temperature for 1 h. Cells were washed with PBS, and nuclei were counterstained with 4,6-diamidino-2-phenylindole. Coverslips were mounted in 70% glycerol, and micrographs were obtained with an Olympus BX50 fluorescence microscope (Tokyo, Japan).

### Flow Cytometry Analysis

A total of 1 × 10^4^ cells/well were seeded in an 6 well containing completed medium for 3 days. At the end of 3 days incubation, the cells were treated by BFP-4 for 24 h. After 24 h incubation, Cells (5 × 10^5^/ml) were incubated in staining buffer (PBS containing 0.5% FBS and 0.1% sodium azide) containing an anti-CD44 and anti-CD51 fluorescein isothiocyanate (FITC) antibody for 30 min on ice. Cells stained with the appropriate isotype-matched were used as negative controls. After staining, cells were fixed with 2% paraformaldehyde and analyzed with an FC500 instrument equipped with Flow ver. 10 (Biosciences, San Diego, CA, United States).

### Western Blot Analysis

Multipotent BMSCs (2 × 10^6^ cells/well) were seeded in a 60Φ cell culture dish and starved by incubation in serum-free DMEM for 6 h. Treated cells were washed with cold PBS and lysed with RIPA lysis buffer (Thermo Scientific, PA, United States) at 4°C for 30 min. The lysates were centrifuged at 13,000×*g* for 15 min, and the supernatants were used as protein samples. The protein concentration was measured using a colorimetric bicinchoninic acid kit (Thermo Scientific, PA, United States) according to the manufacturer’s instructions. Twenty microgram of each cell protein sample was electrophoresed on 10% or 12% SDS-polyacrylamide gel electrophoresis (PAGE) and transferred to polyvinylidene fluoride (PVDF) membranes (Millipore, MA, United States). The membranes were incubated with blocking solution (5% skim milk prepared in Tris-buffered saline) for 1 h. After blocking, membranes were probed with anti-osteocalcin and anti-GAPDH antibodies (Santa Cruz Biotechnology, CA, United States), and then with a horseradish peroxidase-conjugated anti-rabbit or anti-mouse secondary antibody (Santa Cruz Biotechnology) for 2 h. Bands were visualized using an enhanced chemiluminescence detection system (Bio-Rad, CA, United States) and exposed to radiographic film.

### Animal Study

All the animal procedures were carried out in accordance with the guidelines of the Animal Care and Use Committee of Chonnam National University. The animal study was approved by Animal Care and Use Committee of Chonnam National University (Permission number: CNN IACUC-20032). Osteogenically differentiated BFP-4–treated and BMP-7–treated BMSCs were suspended in DMEM at a concentration of 1 × 10^6^ cells/200 µl. Cells were implanted subcutaneously into the right flank of 6-week old male C57BL/6 mice and subjected to point projection digital radiography at 26kV for 3 s using a MX-20 digital microradiography system (Faxitron Bioptics, Lincolnshire, IL, United States). X-ray images were processed using Dicom Works software.

### Statistical Analysis

Results are presented as means and standard deviation (SD). Data were analyzed by a one-way analysis of variance (ANOVA) followed by Duncan’s post-hoc test using SPSS version 11.0 (Chicago, IL, United States). *p* < 0.05 was considered statistically significant.

## Results

### Synthesis of BFP-4

**FIGURE 1:**
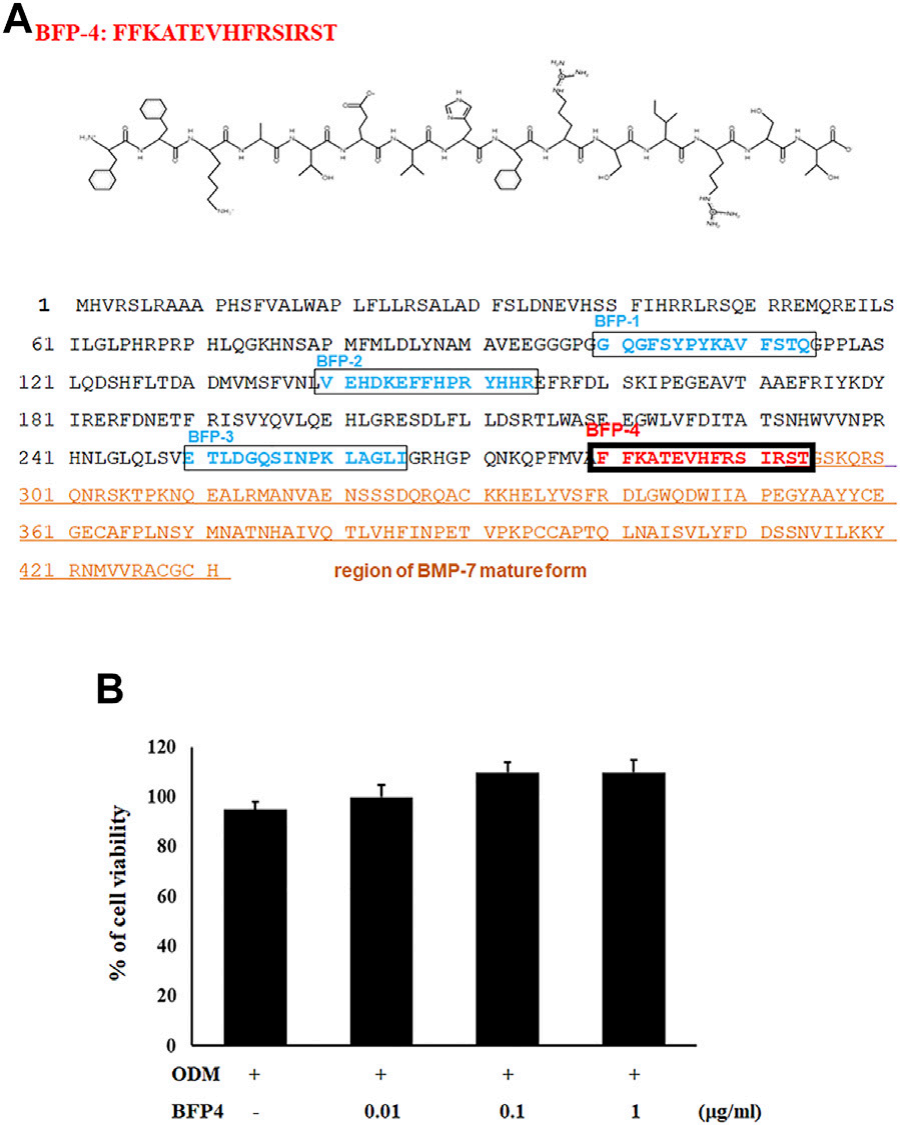
Synthesis of BFP-4 peptide and cell viability. The peptide was synthesized by Fmoc solid-phase peptide synthesis using an automated peptide synthesizer and was purified by reverse-phase high-performance liquid chromatography. The molecular mass of the purified peptide was measured by liquid chromatography/mass spectroscopy **(A)**. BSCs were treated with BFP-4 (0.01 to 1 μg/ml) during the initial phase of osteogenic differentiation. The percentage of viable cells was analyzed by the MTT assay **(B)**. The result is representative of repeated three independent experiments.

We previously reported that the BFP series, which is derived from the immature region of BMP-7, is capable of osteogenic differentiation and bone-regeneration activity. Based on this finding, we focused on other potentially osteogenic peptide sequences in the immature region of BMP-7. We identified a new peptide with the sequence FFKATEVHFRSIRST (Figure 1A), which we called BFP-4. Therefore, we piloted various experiments to determine the osteogenic efficacy of BFP-4.

### BFP-4 Induces Stronger Osteogenic Differentiation of BMSCs Than BMP-7

**FIGURE 2:**
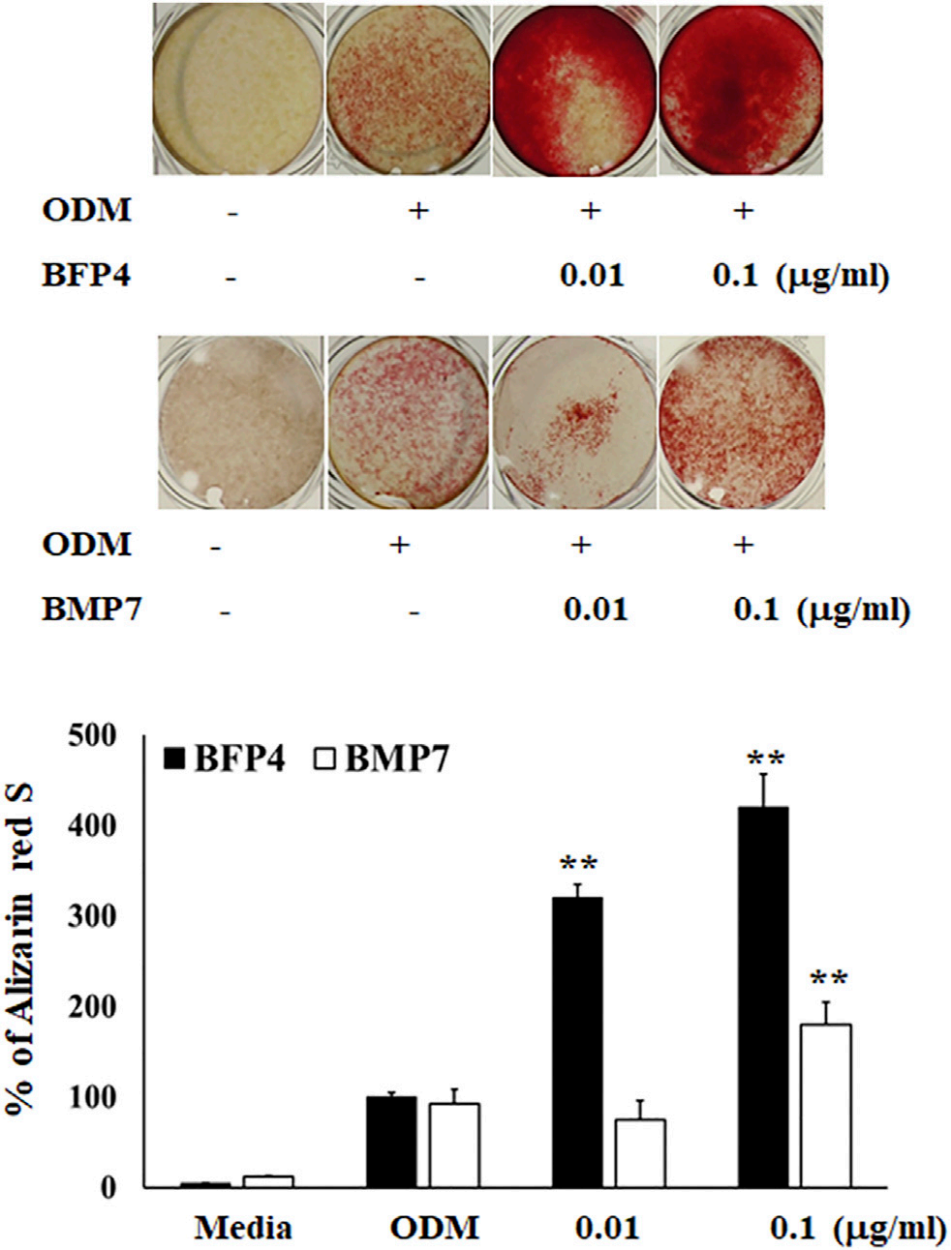
BFP-4 induces osteogenic differentiation BMSCs were treated with a range of concentrations (0.01 and 0.1 μg/ml) of BFP-4 and BMP-7 during the initial phase of osteogenic differentiation and assessed by Alizarin red S staining. Images represent four independent experiments. The result is representative of repeated four independent experiments. Data are mean ± SD of three independent experiments. **p<0.01 compared with the ODM control. Magnification, 20×.

In the first experiment, we assessed the cytotoxicity of BFP-4 (Figure 1B) on multipotent BMSCs by an MTT assay. BFP-4 was not cytotoxic until 1 μg/ml. We next investigated how BFP-4 influences osteogenic differentiation of BMSCs compared with BMP-7. As shown in Figure 2, BFP-4 treatment induced greater osteogenic differentiation of BMSCs than BMP-7 treatment as observed by an Alizarin red S staining assay. Interestingly, treatment with 0.01 and 0.1 μg/ml BPF-4 showed a significantly higher osteogenic differentiation effect than the treatment with 0.01 and 0.1 μg/ml BMP-7. This suggests that BFP-4 could be used as an adjuvant in osteoporotic disease therapy instead of BMP-7.

### BFP-4 Induces Biomarkers of Osteogenic Activity

**FIGURE 3:**
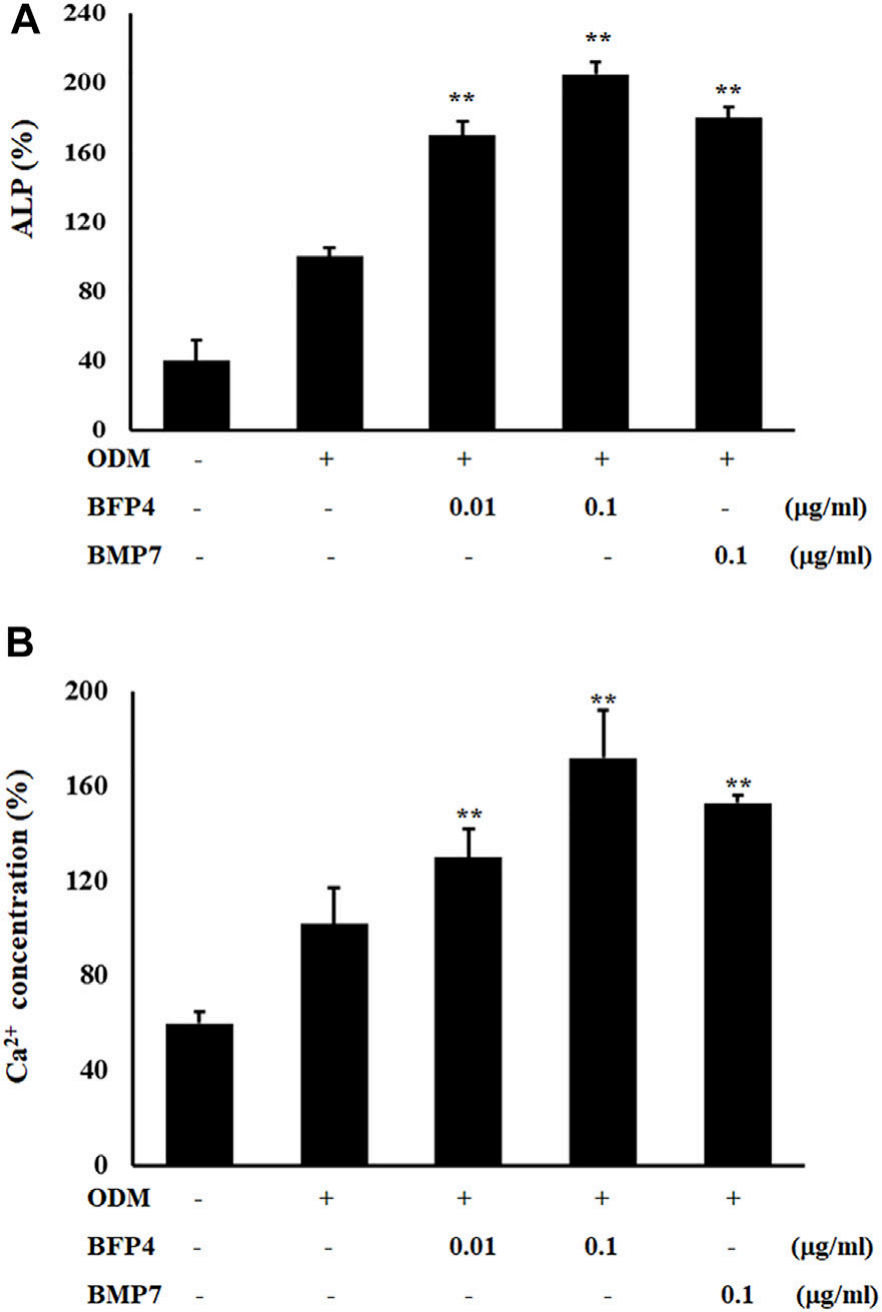
Effect of ALP activity and Ca^2+^ concentration by BFP-4 treatment in BMSCs. Cells were treated with 0.01 or 0.1 μg/ml BFP-4, and 0.1 μg/ml BMP-7 for 24 h. ALP activity **(A)** and Ca^2+^ concentration **(B)** were quantitatively analyzed using a LabAssay™ ALP Assay kit and a calcium detection kit, respectively. Data are mean ± SD of four independent experiments. **p < 0.01 compared with the ODM control.

**FIGURE 4:**
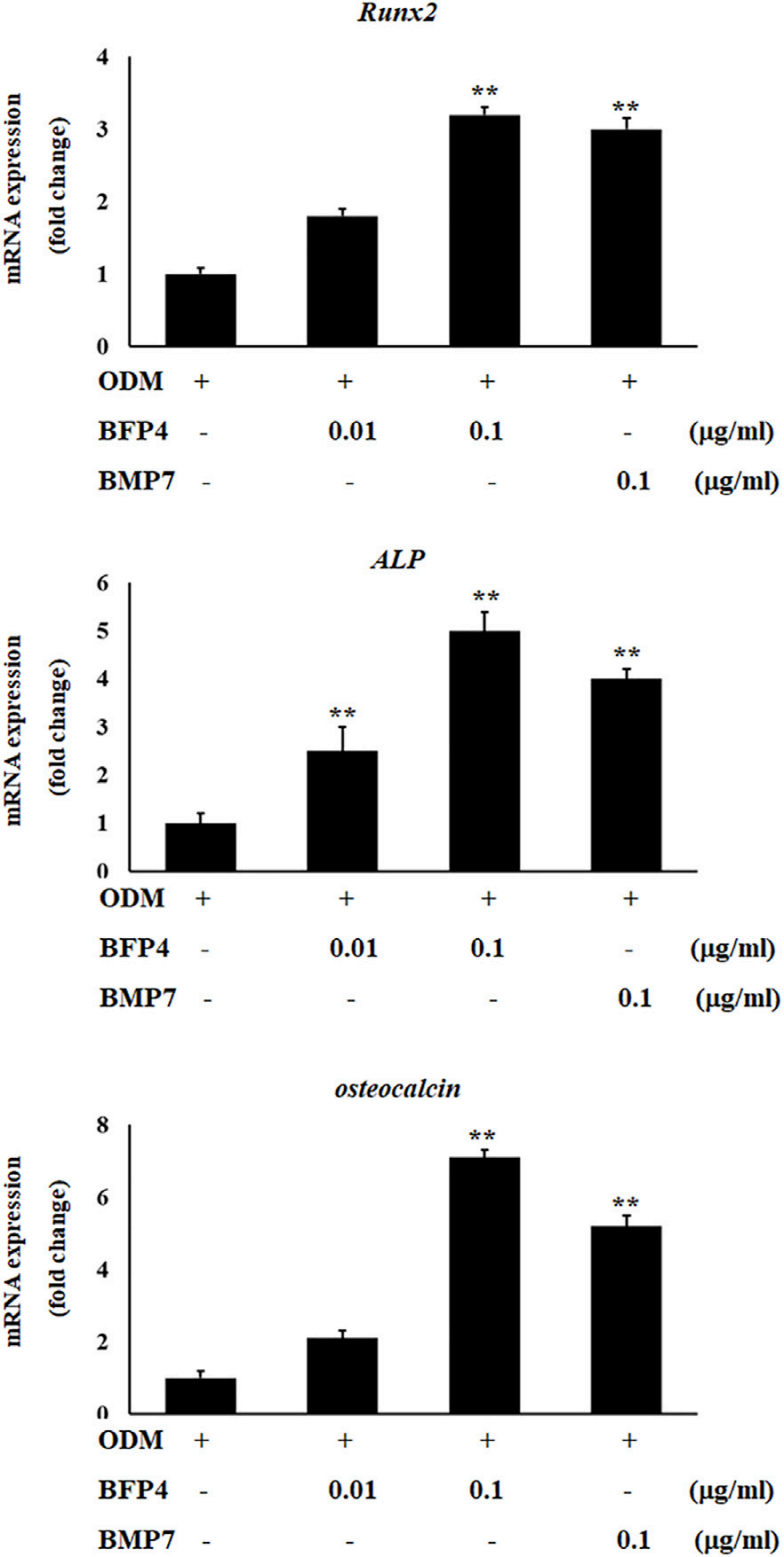
Effect of *ALP, Runx2*, and *osteocalcin* expression by BFP-4 treatment in BMSCs. BMSCs were treated with 0.01–0.1 μg/ml of BFP-4 or 0.1 μg/ml of BMP-7 during osteogenic differentiation. Total RNA was isolated from media only (control) and from cells treated with ODM alone, ODM plus BMP-7, and ODM plus BFP-4. Expression levels were measured by Real-time PCR analysis. The result is representative of repeated three independent experiments. Experimental results were indicated as mean (± SD). **p < 0.01 compared with the ODM control.

Increased ALP activity and calcium concentration are important biomarkers during osteogenic differentiation. Therefore, we investigated whether BFP-4 induces these osteogenic activity biomarkers in BMSCs. ALP activity and Ca^2+^ concentration were significantly increased by BFP-4 treatment in BMSCs (Figures 3A,B). We also determined the effect of BFP-4 on the expression of several genes involved in osteogenesis. Gene expression analysis showed that *ALP, osteocalcin*, and *Runx2* mRNA expression increased during osteogenic differentiation in BFP-4-stimulated cells compared with BMP-7-stimulated cells (Figure 4).

### BFP-4 Induces CD44 and CD51 Expression in During Osteogenic Differentiation

**FIGURE 5:**
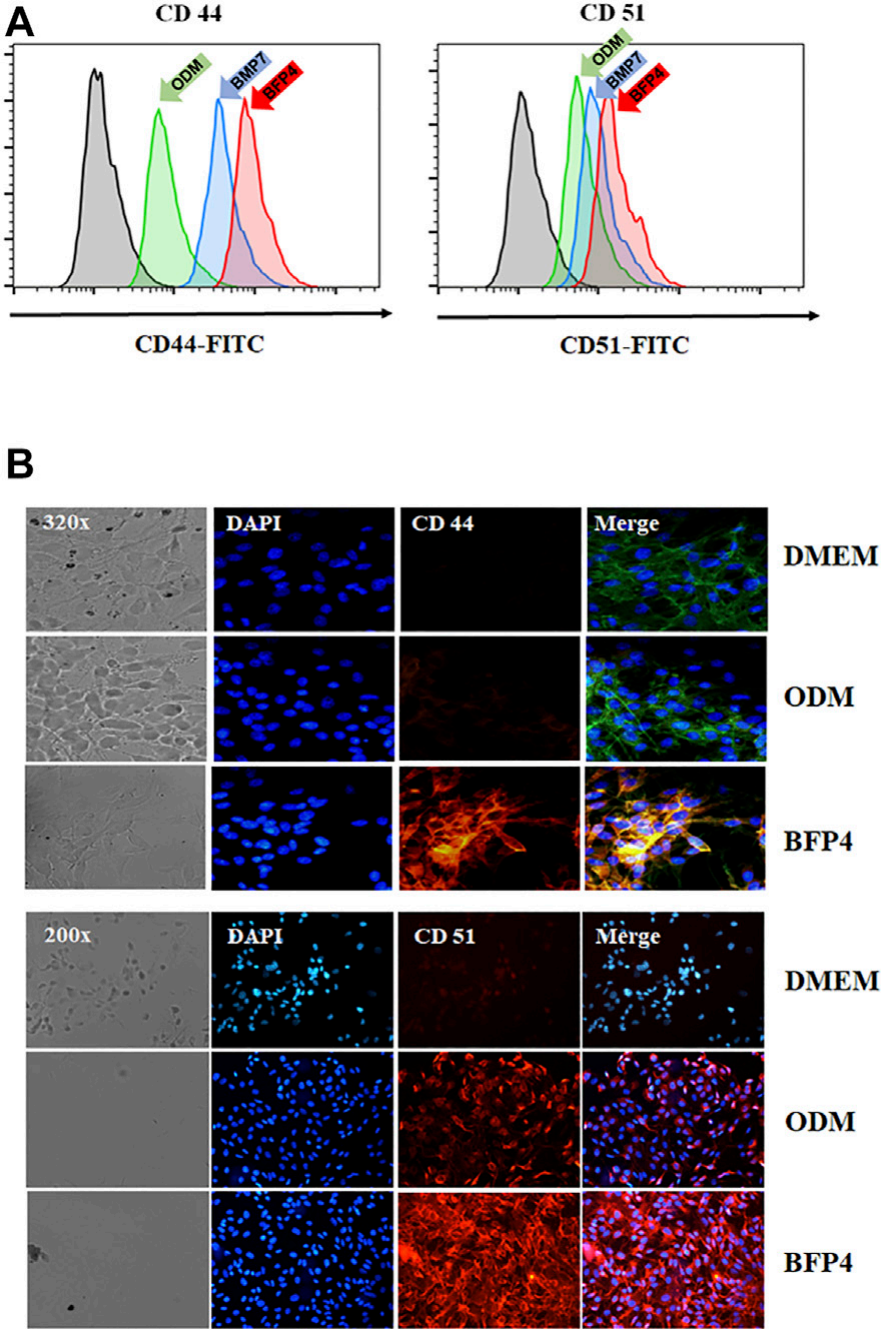
Effect of CD44 and CD51 expression levels by BFP-4 treatment in BMSCs. BMSCs were treated with 0.1 μg/ml BFP-4. After 24 h, CD44 and CD51 expression were measured by flow cytometry (green arrow, ODM only; blue arrow, ODM plus BMP-7; pink arrow, ODM plus BFP4) **(A)**. Immunofluorescence analysis was conducted using anti-CD44 and anti-CD51 antibodies **(B)**. Data represent four independent experiments. Magnification, 400×.

Transmembrane proteins CD44 and CD51 play important roles in various stages of osteogenesis such as osteoblast proliferation and mineralization. To investigate whether BFP-4 induces expression of CD44 and CD51, we measured CD44 and CD51 expression in BFP-4-stimulated multipotent BMSCs by FACS and immunofluorescence analysis. As shown in Figures 5A,B, we found that BFP-4 significantly induced CD44 and CD51 expression in BMSCs. Taken together, these results suggest that the potential osteogenic effects of BFP-4 were manifested through the induction of osteogenic differentiation biomarkers including ALP activity, Ca^2+^ concentration, and the expression of *Runx2, osteocalcin, ALP*, CD44, and CD51.

### BFP-4 Enhances Bone Formation in BFP-4-Treated BMSCs Transplanted Into Mice

**FIGURE 6:**
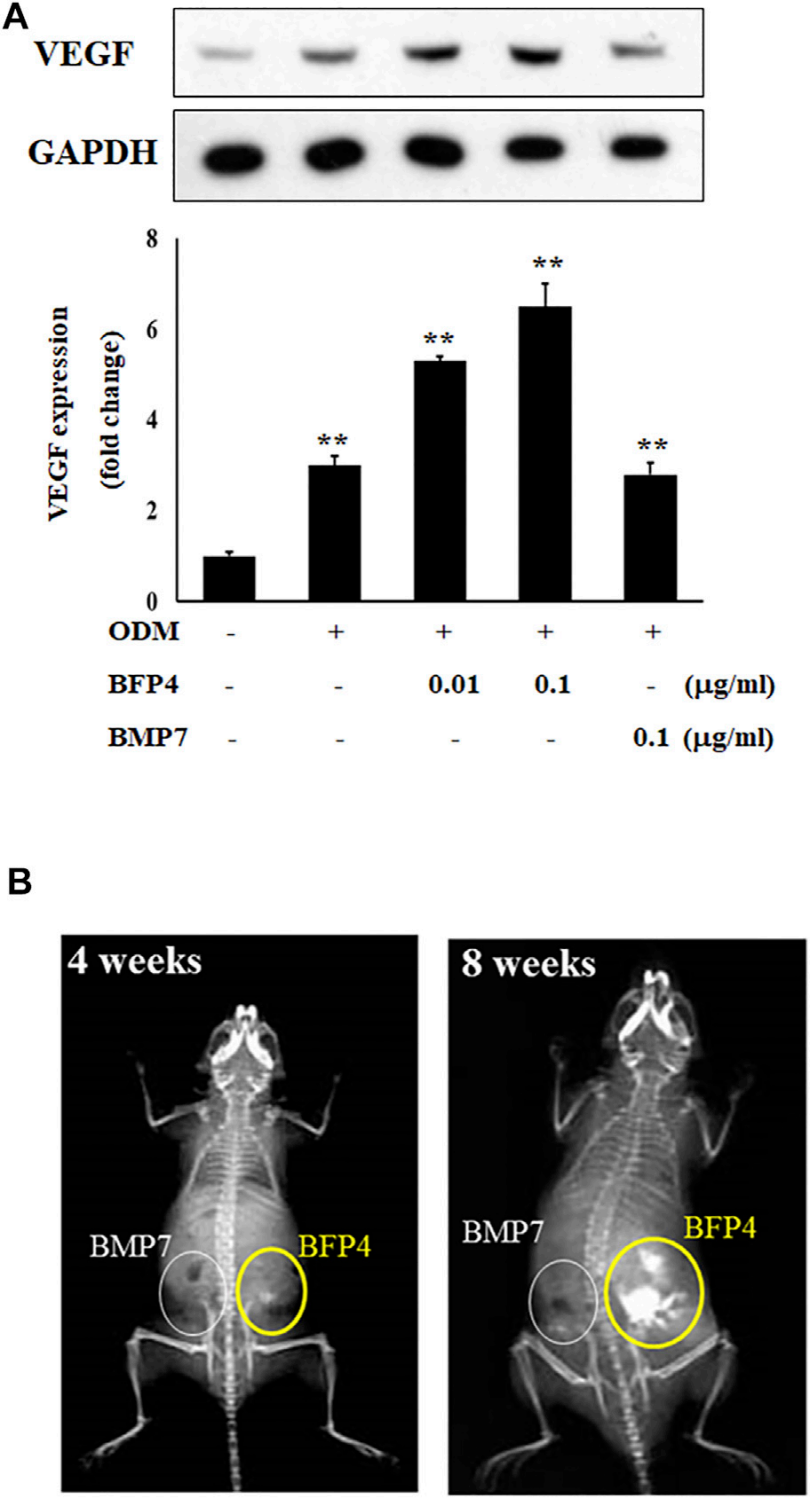
BFP-4 induces VEGF expression and animal bone formation. BFP4-treated cells were lysed, and equal amounts of proteins were separated by SDS-PAGE and transferred to PVDF membranes. Membranes were probed with anti-VEGF and anti-GAPDH antibodies. GAPDH was used as the internal control **(A)**. BMP-7-stimulated and BFP-4-stimulated BMSCs were injected into the left and right flanks of 6-week-old male mice (*n* = 4 mice for each group). The concentrations of BMP-7 and BFP-4 were 0.1 μg/ml. All mice were examined by radiography at 4 and 8 weeks **(B)**. Data represent four independent experiments. **p < 0.01 compared with the ODM control.

VEGF is not only a factor regulating angiogenesis, but also plays critical roles in bone formation and repair (Hu and Olsen, 2016). BMPs have recently been reported to have angiogenic effects, which are important factors in osteogenesis and bone formation. For this reason, we determined the expression of vascular endothelial growth factor (VEGF) to verify that BFP-4 has an angiogenic effect. As shown in Figure 6A, BFP-4 induced VEGF expression in a dose-dependent manner.

Moreover, to determine the potential of BFP-4 for treatment of osteoporotic disease as a therapeutic adjuvant, we next investigated the comparative *in vivo* bone-forming activity of BFP-4 and BMP-7. As shown in Figures 6A,B slight bone formation within approximately 4 weeks was observed in the mice transplanted with the BFP-4-stimulated BMSCs, but not the BMP-7-stimulated BMSCs. In a radiography assay conducted 8 weeks after transplantation, BFP-4-stimulated BMSCs in mice had strongly increased bone formation compared with BMP-7-stimulated BMSCs. Therefore, these results provide new evidence that BFP-4 may be more useful than BMP-7 as an inducer of osteogenesis for bone-related diseases.

## Discussion

BMP was revealed in the 1970s, and demonstrating that these proteins play an important role in osteogenesis and bone formation (Aluganti Narasimhulu and Singla, 2020). Various types of BMPs have been discovered, and each BMP has been reported to have various functions. For example, BMP-2, −4, and −7 have the ability to induces osteogenesis and bone formation, whereas BMP-9 strongly inhibits angiogenesis. In general, BMP-7 is the most commonly known and used for bone formation and regeneration (Li et al., 2010). However, the development of materials related to bone formation and regeneration that are still excellent in efficacy and economical is still required.

The search for new substances that are more economical and effective than BMP-7 and elucidating the mechanisms by which they operate, are important for the development of new therapeutic adjuvants. Previous findings have indicated that mature BMPs are known to have osteogenic effects, but we discovered that BFP peptides of immature regions of BMPs have osteogenic and bone-formation potency. We previously reported on the osteogenic and bone formation efficacy of BFP-1, BFP-2, and BFP-3. The reason for finding these new peptides is to increase the efficiency of bone regeneration-related treatments as well as economic benefits. In the present study, we not only demonstrated the osteogenic efficacy of the newly-discovered BFP-4, but also provided evidence that it has greater bone-forming activity than BMP-7. Furthermore, BFPs, including BFP-4, induce greater osteogenic differentiation and bone formation at the same concentration as BMP-7. As shown in Figure 6, BFP-4-stimulated MSCs have been shown to significantly increase bone formation compared with BMP-7-stimulated BMSCs. Thus it has been demonstrated that BFP-4 is both more effective and more than BMP7.

BMSCs stimulated by BFP-1 and BFP-2 increased levels of ALP, CD44, and CD51 expression as well as Ca^2+^ concentration during osteogenic differentiation (Kim et al., 2012; Kim et al., 2017). Moreover, in the case of BFP-3, the efficacy and molecular mechanism of osteogenic differentiation were elucidated (Lee et al., 2018). It was previously confirmed that BFP-3 not only significantly increased the expression of *osterix* and *Runx2*, which are the major factors of osteogenesis, but also showed osteogenic effect in BMSCs through the regulation of the MAPK signaling pathway. In the present study, we found that BFP-4 induces ALP activity and increases Ca^2+^ concentration in BMSCs (Figure 2). We also determined that the expression of osteogenic factors such as *RUNX2, osteocalcin*, and *ALP* expressions were increased by BFP-4 treatment in BMSCs (Figure 4). In our previous studies as well as our present results, we found that activation of MAPK and NF-κB are important to osteogenesis by BFPs. From these results, bone formation effects on BFPs were confirmed, and specifics of each were identified. Moreover, it was confirmed that the efficacy of BFP-4 found in this study was also excellent. However, little is known about their target receptor or ligand, and that should be the subject of future research.

Moreover, in recent years, the demand for bone growth factors or accelerators to seed 3D scaffolds has increased, because they are used for tissue regeneration in various therapeutic fields. The regeneration of tissue such as cartilage and bone involves seeding cells into a customized polymer scaffold that provides a 3D environment to promote matrix production. Thus, tissue engineering offers the potential to grow osteoblasts quickly in an injectable form, and these injected cell-polymer constructs can ultimately result in the formation of bone-like structures (Chang et al., 2009). The development of various bone growth inducers such as BFP-4 is important because the differentiation efficacy of BMSCs (stimulated by the inducers) can be demonstrated and applied in 3D scaffold tissue engineering technology to help treat various bone-related diseases. Angiogenesis is also important in bone formation, and the angiogenic effect of BMPs has recently been reported. For example, BMP-2 induces angiogenesis in human endothelial progenitor cells (Chen et al., 2018) and bone regeneration (Yang L. et al., 2018). As described above, various BMP proteins have been reported to have angiogenic effects, and are therefore potentially important therapeutic agents. As shown in Figure 6, we found that BFP-4 induces VEGF expression in a dose-dependent manner. Therefore, our results indicated that BFP-4 is an optimal factor for inducing bone formation. In this respect, we believe that the use of peptides is efficient because their synthesis is simple, easy to manipulate, and economical compared to proteins. Although BFPs suggests the possibility of use as a material for bone formation in terms of efficiency and economy, studies on their affinity with receptors or other proteins are needed.

In conclusion, the present study demonstrates that BFP-4 induced greater osteogenic differentiation of BMSCs than BMP-7, both *in vitro* and *in vivo*, and stimulated greater expression of biological markers of osteogenesis than BMP-7. Also, we found that BFP-4 markedly promoted bone formation and also increased the expression of VEGF. Therefore, these results provide new insights into the possible use of BFP-4 as an osteogenic stimulator instead of BMP-7 in clinical trials of bone-related tissue engineering.

## Data Availability

The dataset used and/or analyzed during the current study are available from corresponding author on reasonable request.
